# Influence of Sr deficiency on structural and electrical properties of SrTiO_3_ thin films grown by metal–organic vapor phase epitaxy

**DOI:** 10.1038/s41598-021-87007-2

**Published:** 2021-04-05

**Authors:** Aykut Baki, Julian Stöver, Tobias Schulz, Toni Markurt, Houari Amari, Carsten Richter, Jens Martin, Klaus Irmscher, Martin Albrecht, Jutta Schwarzkopf

**Affiliations:** Leibniz-Institut Für Kristallzüchtung, Max-Born-Straße 2, 12489 Berlin, Germany

**Keywords:** Electronic devices, Information storage

## Abstract

Homoepitaxial growth of SrTiO_3_ thin films on 0.5 wt% niobium doped SrTiO_3_ (100) substrates with high structural perfection was developed using liquid-delivery spin metal–organic vapor phase epitaxy (MOVPE). Exploiting the advantage of adjusting the partial pressures of the individual constituents independently, we tuned the Sr/Ti ratio of the gas phase for realizing, stoichiometric, as well as Sr deficient layers. Quantitative energy dispersive X-ray spectroscopy in a scanning transmission electron microscope confirm Sr deficiency of up to 20% in nominally off-stoichiometrically grown films. Our MOVPE process allows to grow such layers in phase pure state and without extended defect formation. Indications for oxygen deficiency could not be identified. Sr deficient layers exhibit an increased permittivity of *ɛ*_*r*_ = 202 and a larger vertical lattice parameter. Current–voltage characteristics (IVCs) of metal–oxide–semiconductor (Pt/SrTiO_3_/SrTiO_3_:Nb) structures reveal that Sr deficient SrTiO_3_ films show an intrinsic resistive switching with on–off ratios of three orders of magnitude at RT and seven orders of magnitude at 10 K. There is strong evidence that a large deviation from stoichiometry pronounces the resistive switching behavior. IVCs conducted at 10 K indicate a defect-based mechanism instead of mass transport by ion diffusion. This is supported by in-situ STEM investigations that show filaments to form at significant higher voltages than those were resistive switching is observed in our samples.

## Introduction

Strontium-titanate (SrTiO_3_) has widely been investigated due to its interesting physical properties such as high permittivity at room temperature in bulk crystals, ceramics, and thin films^1–3^. While unstrained and undoped SrTiO_3_ is a centrosymmetric material^4^ and paraelectric at room temperature, it can be transferred into a ferroelectric state by the application of strain^5^. By doping with niobium, lanthanum, or samarium, SrTiO_3_ becomes an n-type semiconductor, which may even show superconductivity at very low temperatures as described by Ahadi et al.^6^ Especially in recent years, SrTiO_3_ thin films have gained an increasing interest due to its resistive switching behavior, rendering it a potential candidate for resistive random access memory (ReRAM) applications^7^. However, the underlying physical origin of this effect is still controversial to date. Different models based on Schottky barriers^8^ and charge trapping effects^9^ were discussed to explain the bipolar resistive switching effect, yet the most commonly accepted models for the origin of resistive switching are oxygen migration models^7,10–14^. By application of a high bias pulse, conductive filaments of oxygen vacancies are formed and ruptured by diffusion processes, which leads to a low resistive state (LRS) and high resistive state (HRS), respectively. The impact of cation deficiency^15,16^ on soft-forming processes was investigated on thin films grown by pulsed laser deposition (PLD)^17,18^.

Growth of SrTiO_3_ thin films by different techniques has often been reported in literature. The most frequently used method is pulsed laser deposition (PLD), but also (hybrid) molecular beam epitaxy (MBE)^19,20^, sol–gel^21^, and metal–organic chemical vapor deposition (MOCVD)^22–26^ techniques have been utilized. While in the PLD process film composition and structure are determined by an interplay of different growth parameters, like laser fluence, geometry, background pressure and target composition, MBE and MOCVD offer the possibility to independently control growth parameters determining the film composition, especially the partial pressures of all components in the gas phase. In MOCVD of SrTiO_3_ thin films, the Sr, Ti, and O content in the films is provided by precise adjustment of the respective precursor supply fluxes. Further advantages of MOCVD are that film growth takes place near thermodynamic equilibrium and at high oxygen partial pressure. Typical values are in the range of tens of mbar, which is about 3 orders of magnitude higher than in PLD and 9 orders higher than in MBE. The combination of these characteristics of the MOCVD method enables the growth of epitaxial films of high structural perfection^27^ with stoichiometric, as well as intentional off-stoichiometric composition while maintaining the perovskite structure without foreign phases. Defined cation deviations from stoichiometry with almost full oxygen-site occupancy can be independently realized with this growth method. This provides the possibility to determine the physical properties of complex perovskites as a function of intentionally incorporated defects^18,28^.

So far, the growth of SrTiO_3_ by MOCVD has only been demonstrated on platinum covered silicon substrates and resulted in polycrystalline layers^22–26^. Due to this polycrystalline nature, i.e. a high density of structural defects, a deeper understanding of the physical properties has been impeded. These results demonstrate, nonetheless, the unique feature of this equilibrium deposition method to grow SrTiO_3_ thin films with high deviations from stoichiometric composition without the formation of secondary phases.

In this paper, we demonstrate the appropriate growth regime for deposition of phase-pure SrTiO_3_ epitaxial thin films by MOCVD, which we will denote as metal–organic vapor phase epitaxy (MOVPE) in the following.

As a result of these investigations, we were able to realize coherent, homoepitaxial growth of phase pure SrTiO_3_ thin films on 0.5 wt% Nb doped SrTiO_3_ substrates. By independent control of Sr and Ti fluxes into the reaction chamber, we have precisely adjusted the Sr/Ti ratio in the gas phase and thus in the films, which allows us to systematically study the impact of Sr/Ti ratio on the structural and (di)electrical properties. Thereby, we found an increasing dielectric permittivity and a pronounced intrinsic resistive switching behavior at high Sr deficiency.

We reveal that the underlying point defects in the film are related to deliberately introduced Sr deficiency. Further, we report on intrinsic resistive switching behavior even at low temperatures of 10 K, where ionic diffusion processes should be strongly suppressed. Hence, resistive switching in non-stoichiometric films (strong Sr deficiency of 20%) shows an atypical evolution compared to reported resistive switching devices based on SrTiO_3_ materials, providing evidence of defect assisted transition instead of a mechanism based on mass transport.

## Results and discussion

Section A is focusing on the growth, stoichiometry and characterization of the defects present in the films, whereas section B is dealing with the impact of Sr deficiency on the permittivity and resistive switching. In Section C, *in-situ* STEM investigations are presented.

### Growth and stoichiometry control of SrTiO_3_ by MOVPE

**Figure 1:**
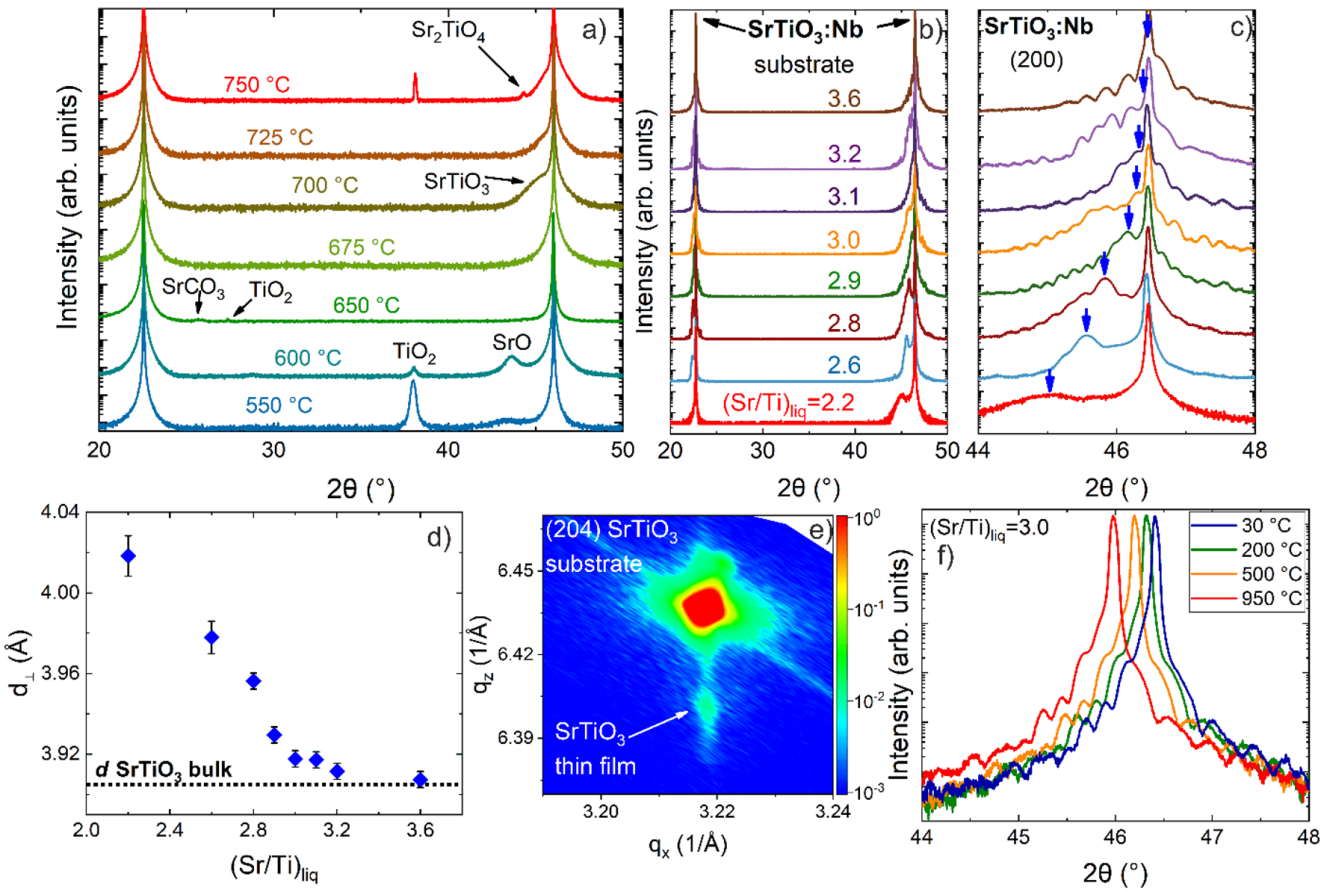
HRXRD patterns of (**a**) thin films grown under various substrate temperatures T_s_ from 550 °C to 750 °C on DyScO_3_ (110), and (**b**) SrTiO_3_ films grown with various precursors concentration ratios in the liquid sources (Sr/Ti)_liq_ grown on SrTiO_3_:Nb (100) in a 2θ range between 20° and 50°. (**c**) The same HRXRD scans from (**b**) with higher magnification in the vicinity of the SrTiO_3_:Nb (200) substrate peak (the film peak position is illustrated by blue arrows) with increasing (Sr/Ti)_liq_. (**d**) Vertical lattice parameter *d*_⊥_ as a function of (Sr/Ti)_liq_. (**e**) Reciprocal space maps of the films with (Sr/Ti)_liq_ = 2.6 in the vicinity of the (204) Bragg peak of bulk SrTiO_3_. (**f**) In-situ HRXRD patterns at different temperatures of post-annealed SrTiO_3_ sample with (Sr/Ti)_liq_ = 3.0 in pure oxygen at ambient pressure up to 950 °C.

In order to determine the appropriate substrate growth temperature (T_s_) for achieving single-phase SrTiO_3_ growth, depositions were performed from 550 to 750 °C. The thin films were grown on DyScO_3_ (110) substrates using a (Sr/Ti)_liq_ = 1 with an absolute Sr concentration of c_Sr(tmhd)2_ = 0.025 mol/l. DyScO_3_ was chosen here as substrate to distinguish film and substrate peaks in high-resolution X-ray diffraction (HRXRD). Figure 1a shows 2θ/ω scans of samples grown under various substrate temperatures T_s_.

For growth temperatures below 675 °C, additional Bragg reflection peaks occur at 2θ of about 27°, 32°, 38°, and 43°, which are attributed to the single oxide contributions TiO_2_ (103), SrO (111), TiO_2_ (203), and SrO (104), respectively^29,30^. Additionally, a SrCO_3_ (111) phase (2θ ≈ 26°) is formed at 650 °C^31^. From 675 to 725 °C, single-phase SrTiO_3_ with (100) surface orientation was observed, whereas at a temperatures of 750 °C additional Ruddlesden–Popper (RP) phases like Sr_2_TiO_4_ and a TiO_2_ phase are present. The vertical lattice parameter of the SrTiO_3_ thin films occurs in the vicinity of the DyScO_3_ peak and thus at a lower 2θ angle compared to an unstrained, stoichiometric SrTiO_3_. This is attributed to an off-stoichiometry in the films, which will be discussed in more detail below. This temperature dependence of phase formation agrees with literature data and is reasoned by the standard formation enthalpies^32^. Single oxides in general have lower formation enthalpies than their corresponding complex oxides and hence are formed at lower T_s_. When T_s_ is increased to 750 °C, the energy provided is sufficient to form RP phases, which are known to have even higher standard formation enthalpies than SrTiO_3_^32^. Thus, in order to obtain single-phase SrTiO_3_ without the formation of single oxides, carbonates, and RP phases, T_s_ should be adjusted in the range of 675 °C to 725 °C.

Next, we grew a series of samples on 0.5 wt% Nb-doped SrTiO_3_ (100) substrates using a fixed T_s_ of 710 °C. Here, the (Sr/Ti)_liq_ ratio in the source materials was varied from 2.0 to 3.6. By controlling the Sr and Ti gas flows, the corresponding partial pressures—and particularly the ratio of the partial pressures—are precisely adjusted in the gas phase and consequently, the cation composition in the layer can be modified in a targeted way. Figure 1b shows the corresponding HRXRD patterns of the thin films with the film reflection emerging as small shoulders at the low angle side of the substrate peak. As achieved for the series grown on DyScO_3_, no parasitic phases like SrO, SrCO_3_, TiO_2_, Sr_*n*+1_Ti_*n*_O_3*n*+1_ (*n* = 1, 2…) appear for this growth temperature, demonstrating phase pure (100) SrTiO_3_ epitaxial films for all applied (Sr/Ti)_liq_ ratios.

In Fig. 1c, a higher angular resolution of the HRXRD pattern around the (200) substrate peak is presented. When the (Sr/Ti)_liq_ ratios were below 3.2, the film peak occurs at lower 2θ angles as compared to the SrTiO_3_ substrate peak. Furthermore, for (Sr/Ti)_liq_ > 2.6, occurrence of thickness fringes can clearly be observed, which indicates a high crystalline quality of the film including smooth interfaces and surfaces. However, the thickness fringes in the HRXRD patterns are overlaid with irregular oscillations for almost all films. As we will discuss later in conjunction with the STEM investigation, these oscillations are related to a fluctuating defect distribution along the growth axis and hamper the fitting of the HRXRD pattern, which assumes a single homogenous layer. Rather, a stack of at least two films each with a different vertical lattice parameter is required to match measurement and simulation. For detailed insights into the HRXRD pattern simulations, see section HRXRD simulation in the Supplemental.

The evaluation of all HRXRD data indicates that the film peak is approaching the position of SrTiO_3_ bulk substrate with increasing (Sr/Ti)_liq_ ratio up to 3.6. From the angular difference of the Bragg reflection of the film and the substrate, the vertical lattice parameters *d*_*⊥*_ of all samples with 2.0 ≤ (Sr/Ti)_liq_ ≤ 3.6 are calculated and plotted in Fig. 1d. The plot reveals a continuous increase of the vertical lattice parameters *d*_*⊥*_ with decreasing (Sr/Ti)_liq_ ratios. We attribute the enlarged lattice parameters of the films for (Sr/Ti)_liq_ < 3.6 to an off-stoichiometry and a resulting compressive in-plane lattice strain in the films. Only for (Sr/Ti)_liq_ = 3.6, the film lattice parameter matches the bulk lattice parameter of *d*_*SrTiO3*_ = 3.905 Å^33^, suggesting stoichiometric film composition and a strain-less film. The correlation between the increased vertical lattice parameter in our MOVPE thin film and the deviation from stoichiometric composition qualitatively agrees with the results from other deposition techniques^34–37^. However, the observed deviations of the vertical lattice parameter in our MOVPE films from the value for unstrained, stoichiometric SrTiO_3_ is much larger than for SrTiO_3_ thin films grown by PLD or MBE methods as summarized by Brooks et al.^19^ indicating that we can achieve much higher off-stoichiometric conditions without occurrence of secondary phases. This is attributed to the fact that MOVPE is operated close to thermodynamic equilibrium: by the application of suitable substrate temperatures the formation of secondary phases is suppressed. This possibility to stabilize huge deviations from stoichiometric composition gives MOVPE an unique characteristic in comparison to all the other deposition methods.

In order to verify the epitaxial relation between film and substrate, we performed reciprocal space maps (RSM) for a non-stoichiometric film with (Sr/Ti)_liq_ = 2.6 in the vicinity of the asymmetric (204) Bragg reflection of bulk SrTiO_3_ (see Fig. 1e). The appearance of substrate and film peaks at the same in-plane reciprocal lattice parameter indicates pseudomorphic epitaxial film growth on the SrTiO_3_ substrate.

To rule out that an oxygen deficiency is responsible for the systematic change of the lattice parameter in the nominally Sr deficient layers in the as-grown-state, we performed in-situ 2θ/ω HRXRD scans while annealing the samples in pure oxygen atmosphere up to 950 °C. HRXRD patterns were recorded for a sample grown at (Sr/Ti)_liq_ = 3.0 at 30 °C, 200 °C, 500 °C and from 500 to 950 °C in 25 K intervals with one hour dwell time at each stage, as shown in Fig. 1f for selected temperatures. After completing the described annealing cycle, another 2θ/ω HRXRD scan was again recorded at 30 °C. Neither the film peak position nor the shape or distance of the thickness oscillations changed during the annealing and the subsequent cool down to 30 °C. The observed shift on 2θ axis is due to thermal expansion of the sample during heating up and is reproducible. On the basis of this result, we conclude that the irregularities in oscillations as well as the peak shift to lower 2θ angles cannot be predominately explained by the presence of oxygen vacancies, since their density should be reduced by this post growth annealing in pure oxygen atmosphere, which has not been observed here^38^. The systematic increase of the vertical lattice parameter with decreasing Sr/Ti in the gas phase (Fig. 1d) must therefore basically be attributed to Sr deficiency in the as-grown-state of the SrTiO_3_ films. As mentioned above, all HRXRD patterns except that of the stoichiometric film exhibit irregular oscillations which means that the film contribution cannot be simulated by one single composition, but stacks with different vertical lattice parameters are necessary. From this observation we infer that the film composition is inhomogeneous along the growth axis. Only films grown with a (Sr/Ti)_liq_ = 3.6 reach the SrTiO_3_ bulk lattice constant, implying a stoichiometric composition, and exhibit regular thickness oscillations.

**Figure 2:**
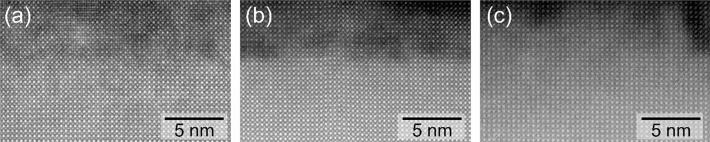
STEM HAADF images for samples with Sr/Ti ratio of (**a**) 2.6, (**b**) 3.2 and (**c**) 3.6 for a camera length of 196 mm.

For a microscopic study of the structural properties and the stoichiometry, we investigated nominally Sr deficient layers grown with a (Sr/Ti)_liq_ ratio of 2.6, 3.2, and the stoichiometric layer with a (Sr/Ti)_liq_ ratio of 3.6 by TEM. The corresponding high-resolution scanning transmission electron microscopy (STEM) high angle annular dark field (HAADF) images are displayed in Fig. 2a–c, respectively. For the sake of comparability, the HAADF intensities in the region of the SrTiO_3_ substrate were normalized to unity. All STEM-HAADF images confirm the formation of single-crystalline and coherently grown SrTiO_3_ films without the presence of foreign phases, in agreement with the HRXRD observations. Furthermore, no extended defects or misfit dislocations could be observed by STEM. For the given HAADF imaging conditions (camera length of 196 mm), layers with (Sr/Ti)_liq_ ratio of 2.6 and 3.2 generally appear considerably darker as compared to the SrTiO_3_ substrate with a cloudy contrast. In comparison, films with a (Sr/Ti)_liq_ ratio of 3.6 only show a faint contrast with respect to the substrate making the film-substrate interface indistinguishable in some regions. Under these imaging conditions, the intensity of the atomic column reflects the mean atomic number (Z-contrast). Therefore, we conclude that films with a (Sr/Ti)_liq_ ratio of 2.6 and 3.2 exhibit a reduced mean atomic number as compared to the substrate, which is in-line with a Sr deficiency in the layer as intended. In contrast, the faint contrast to the substrate of the film with a (Sr/Ti)_liq_ ratio of 3.6 shows near stoichiometry.

Notably, the STEM-HAADF contrast varies across the epitaxial layers of the films in Fig. 2, indicating a spatially inhomogeneous distribution of the underlying defects. In particular, we notice a composition variation along the growth direction. This was also reflected in the different contributions to the HRXRD patterns, such as the smearing of the film peak and the superposition of several interfering patterns (Details see Supplemental). Moreover, for the lowest (Sr/Ti)_liq_ ratio of 2.6, we often observe a stacking defect in the STEM-HAADF images, whereby the SrTiO_3_ unit cell is laterally shifted by a half-unit cell perpendicular to the beam direction. This defect is similar to an anti-site domain boundary and is discussed in detailed in the Supplemental section.

**Figure 3:**
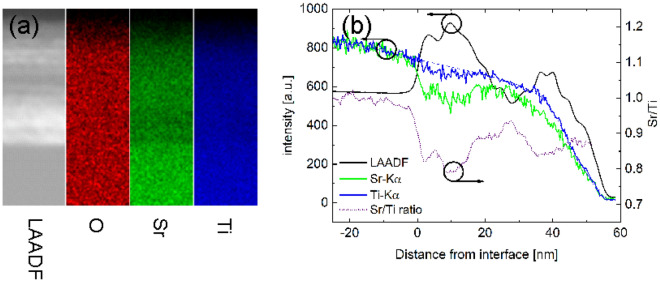
(**a**) STEM LAADF and the corresponding EDX maps of O, Sr and Ti of an off-stoichiometric sample with a (Sr/Ti)_liq_ ratio of 3.2. (**b**) Quantitative analysis of the LAADF intensity (black solid curve), Sr EDX intensity (green), Ti EDX intensity (blue), as well as the ratio of the Sr/Ti EDX signal along the growth direction (purple).

To further corroborate the Sr deficiency in the layer, we carried out additional STEM energy dispersive X-ray (STEM-EDX) measurements on an off-stoichiometric film grown with (Sr/Ti)_liq_ ratio of 3.2. Figure 3a exhibits the color-coded EDX maps of the Sr, Ti and O EDX signal, as well as the corresponding STEM low-angle annular dark field (LAADF) intensity (camera length 300 mm). While the Ti and O signals reveal no apparent change between the layer and the substrate region, the Sr signal in the MOVPE layer is substantially lower, which is particularly pronounced close to the interface. The STEM-LAADF map, which is sensitive under these conditions to diffuse scattering from strong atomic displacements along the beam direction, clearly correlates with the Sr EDX intensity. This implies that Sr vacancy and/or their related complexes are responsible for the observed lattice distortions. To quantify the Sr deficiency, Fig. 3b displays the horizontally averaged EDX signals of Sr (green line) and Ti (blue line) along the growth direction. We have quantified the precision of the EDX measurements by determining the 95% confidence interval of the Sr/Ti ratio values measured in the stoichiometric substrate. The spreading of the values determined in this way is ± 1.2%, which corresponds to the measurement error. In the stoichiometric substrate region, which serves as reference, the Sr and Ti signals are practically identical. Within the epitaxial layer, the Ti as well as the Sr signal decrease in intensity, with the latter being much more pronounced. To quantify the decrease of Sr, Fig. 3b displays the ratio between the Sr and Ti signal (purple line), revealing Sr deficiency up to around 20% in the SrTiO_3_ film close to the substrate interface. Regarding the slightly smaller Ti signal in the MOVPE layer, we believe that this effect is related to the strong atomic displacements, which are locally changing the channeling conditions and thus the corresponding Ti EDX signal. Nevertheless, if there is a lower Ti signal in the layer, its contribution is negligibly small. Towards the surface, the Sr content increases, which essentially results in an unintentional sub layer structure. We have cross-checked such substantial Sr deficiencies by means of off-axis STEM-HAADF, yielding a comparable outcome (see Supplemental).

### Electrical properties of Sr deficient layers

**Figure 4:**
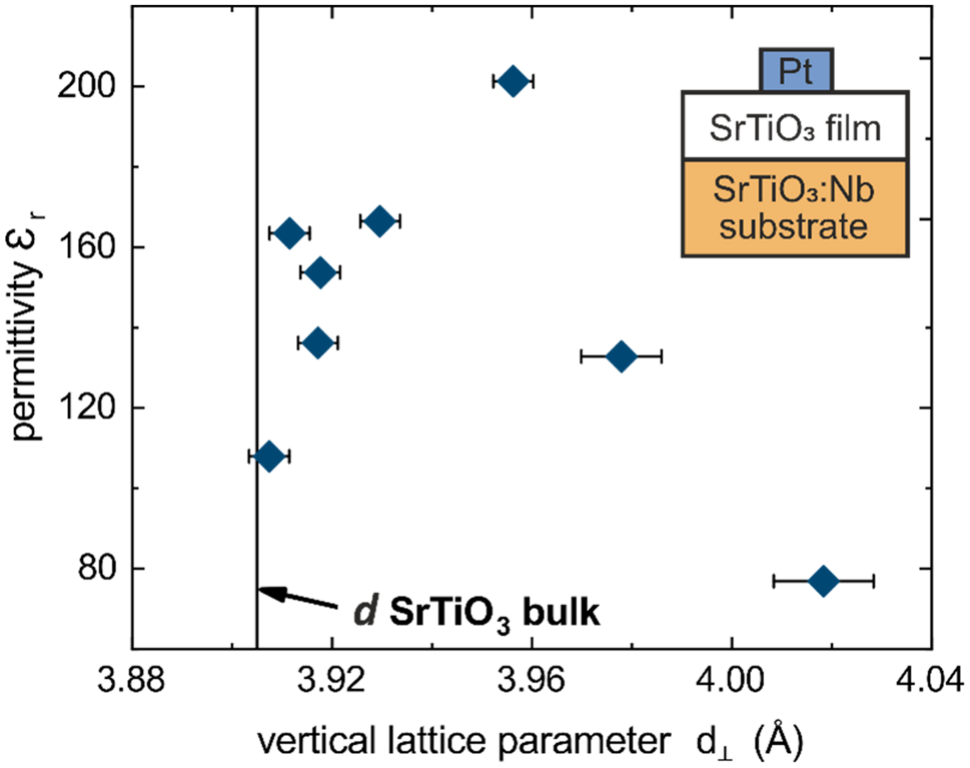
Permittivity *ε*_*r*_ at RT of as grown MOVPE SrTiO_3_ thin films as a function of the vertical lattice parameter *d*_*⊥*_. Inset figure shows the MOS-structure used for this measurement composed of SrTiO_3_:Nb, MOVPE SrTiO_3_ thin film and Pt top contact. Error bars of the permittivity measurement are within the symbol size and results mainly from the uncertainties in contact sizes.

In this section, the influence of the Sr deficiency on the (di)electrical properties of the SrTiO_3_ thin films is investigated. For this purpose, the MOVPE layers were measured in the metal–oxide–semiconductor (MOS) structure described in the methods section and schematically shown in the inset of Fig. 4. All layers were initially insulating for RT and 10 K measurements (> GΩ at a bias voltage of 0.1 V). The relative permittivity was measured at room temperature and showed no dependence on contact size. In Fig. 4, the permittivity is shown versus the vertical lattice parameter. The permittivity continuously increases in the range between *d*_*⊥* _= 3.907 Å to *d*_*⊥* _= 3.956 Å from 110 to 202 and then again decreases for samples with higher vertical lattice parameter. The film with *d*_*⊥*_ = 3.956 Å exhibits the highest permittivity, even higher than the film with stoichiometric composition (*d*_*⊥*_ = 3.907 Å). This evolution agrees qualitatively with literature data for films grown by PLD, MOCVD and sol–gel proccesses^2^ and is understood in terms of polar nanoregions and lattice distortions breaking the paraelectric cubic symmetry of stoichiometric SrTiO_3_^39–41^. Furthermore, it can be deduced that the permittivity cannot be regarded as a fixed material property since it critically depends on the amount of Sr deficiency.

For the electrical characterization, current–voltage characteristics (IVCs) were measured first at room temperature in the voltage range of − 4 V ≤ V ≤ 3 V. The polarity is chosen as it is commonly done in literature with the back-contact on substrate side as common ground and the applied bias at the top-contact. These cycles were reproducible for several repetitions for both kind of samples.

**Figure 5:**
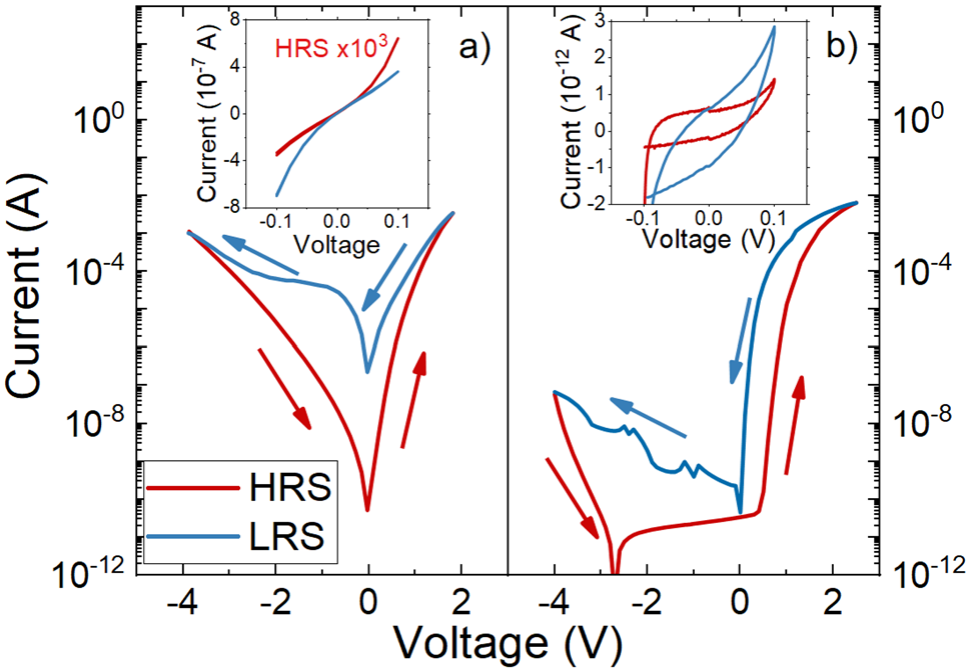
IV curves for samples with two different values of (**a**) (Sr/Ti)_liq_ = 3.2, and (**b**) 3.6. The IV curves are measured in the range of − 4 V to + 3 V showing two different states of resistivity. The upwards measurement in HRS is shown as red line, while the downwards measurement is shown as blue line. The probing of the state (HRS/LRS) is shown in each plot as inset.

Sr deficient samples with (Sr/Ti)_liq_ = 3.2 showed the typical hysteresis behavior for intrinsic resistive switching (see Fig. 5a). Once the LRS or the HRS is enabled, it is possible to probe each state within the bias range of − 0.1 V ≤ V ≤ 0.1 V without any change of resistivity (insets Fig. 5a). The on–off ratio defined as I_LRS_/I_HRS_ at 0.01 V is about 10^3^ for the off-stoichiometric sample.

In contrast, for the stoichiometric thin films with (Sr/Ti)_liq_ = 3.6, stable resistive switching could not be observed in this applied voltage range (see Fig. 5b). The resistivity at read-out voltage of 0.1 V stays at a high level, similar to the initial resistivity of an as grown sample.

At bias voltages larger than 0.5 V a significant current increase can be observed. At such high field strengths of up to 1 MV/cm, injected charge carriers into the film play a role in electrical transport of the thin film, e.g. by Schottky^42^ or Poole–Frenkel emission^43^ A detailed analysis of the electrical transport is beyond the scope of this work and will be given elsewhere.

Next, current–voltage measurements of non-stoichiometric films with (Sr/Ti)_liq_ = 3.2 were performed at low temperature using a closed-cycle refrigerator (T = 10 K, He background pressure = 10^–6^ mbar) to demonstrate resistive switching. At low temperature, the switching voltage is more clearly visible and remains with about 1.5 V essentially unchanged compared to room temperature.

**Figure 6:**
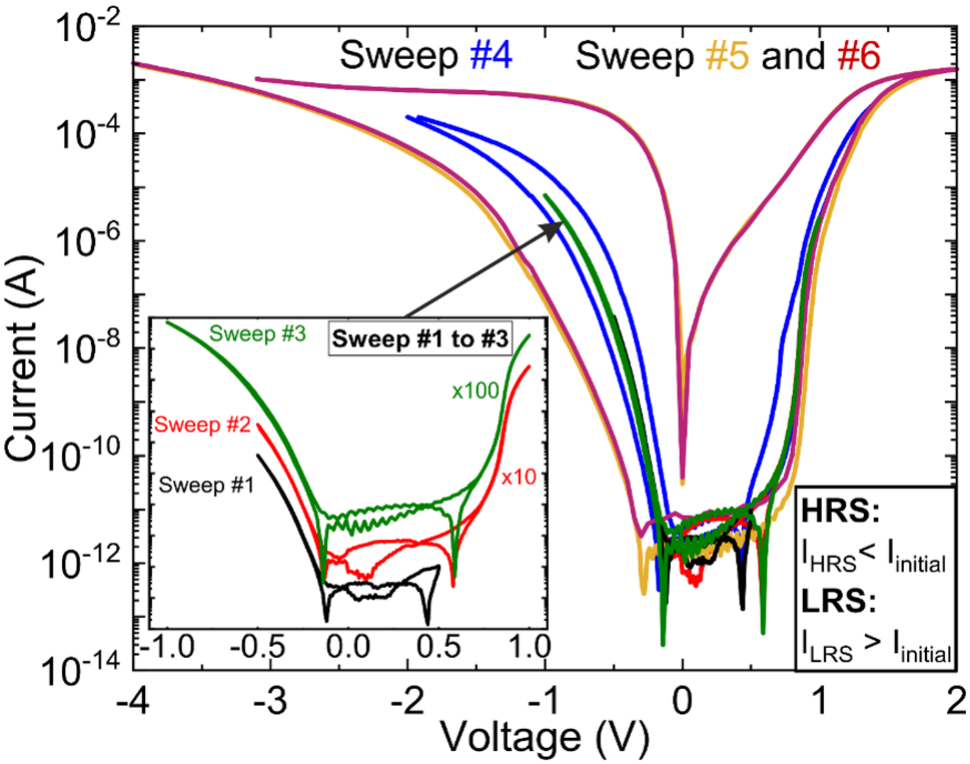
Current voltage measurements at 10 K for SrTiO_3_ thin film with (Sr/Ti)_liq_ = 3.2. The voltage range was gradually increased until resistive switching occurs (Sweep #1 to #6). The curves of sweep #1 to #3 are shown vertically shifted to each other by a factor of 10 in the inset. (Partial) switching occurs for V > 1.5 V.

Another crucial observation is the soft forming process at low temperature. For this, the voltage range was gradually increased until intrinsic resistive switching is introduced. Within the first three sweeps (sweep #1 to #3 in inset of Fig. 6), the voltage is stepwise increased to the threshold voltage of − 1 V. At this point, no resistive switching is observed. Only starting from sweep #4 (− 2 V to 1.5 V), partial resistive switching could be observed. By further increase of the voltage (sweep #5), the on–off ratio increases to about seven order of magnitude. After reaching the hysteresis of sweep #5 (− 4 V to 2 V) intrinsic resistive switching is stable for all following cycles, as illustrated by sweep #6. As shown, the hysteresis evolved without forming pulse and gradually without any abrupt change. Remarkably, the HRS for IVCs displaying (partial) switching (#4 to #6) show a higher resistivity compared to the IV measurements below the threshold (#1 to #3). This is especially visible for negative bias. This observation is in strong contrast to the soft-forming process in Sr deficient resistive switching materials, as described by Lenser et al.^13^, and Stille et al.^44^ There, the overall conductivity of the sample increases since oxygen vacancies are incorporated during the first sweep, and hence implement additional charge carriers^13,44^. In our particular case of Sr deficient MOVPE thin films, we observe the opposite, thereby providing evidence of an alternative mechanism for resistive switching, which is not based on ion diffusion. The contribution of ion diffusion can be neglected for our IV measurement conditions at 10 K. In addition, we can exclude intrinsic resistive switching based on antiphase boundaries as shown by Hensling et al.^45^ The existence of antiphase boundaries could not be verified by TEM. Further, due to our Ti-rich growth regime, Ruddlesden–Popper phases are very unlikely to be formed^46^. Besides, in resistive switching based on antiphase boundary, the HRS should again show the same resistivity like the initial state, but not a higher one like it is observed for our MOVPE thin films. These unique features of the MOVPE grown SrTiO_3_ thin films, which are inconsistent with the established models based on oxygen vacancy drift, require further work to be published in a follow up publication.

### In-situ scanning transmission electron microscopy (STEM) measurements

To get insight into potential structural modifications that cause resistive switching in our Sr deficient SrTiO_3_ samples we performed in-situ STEM measurements. For these measurements, a thin lamella with a thickness of ~ 100 nm is used that is prepared by focused ion beam (FIB). Pt deposition by electron beam evaporation was used for the front side contact. In order to investigate resistive switching behavior of the device under electrical bias, we applied a number of voltage cycles and increased the voltage after each step (± 3 V, ± 5 V and ± 7 V), through which the device was set into the ON or OFF-state, respectively.

**Figure 7:**
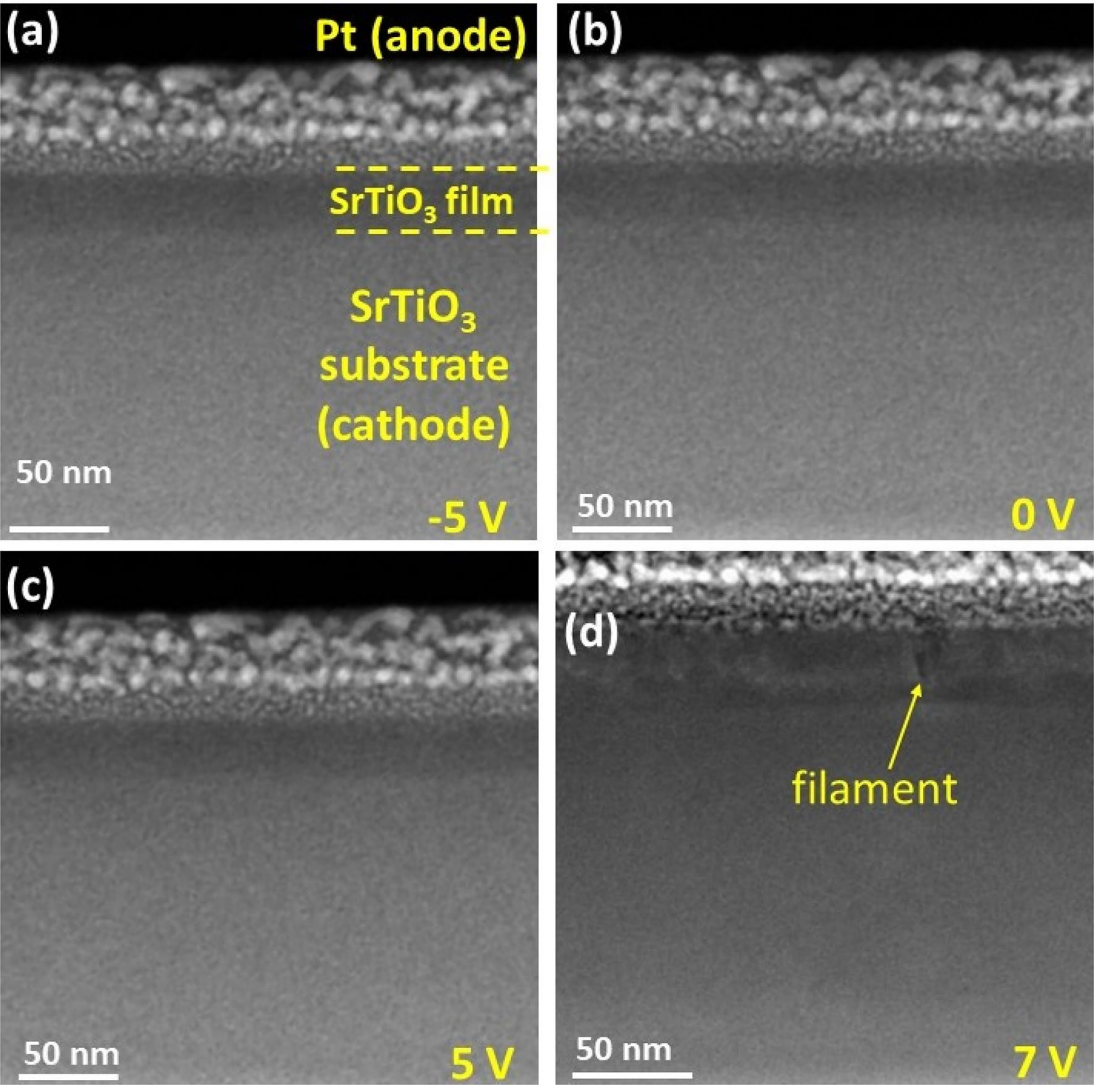
STEM-HAADF images of the device cross-section, after a sweep to (**a**) − 5 V, (**b**) 0 V, (**c**) 5 V and (**d**) 7 V using a current compliance of 30 µA. Dashed lines in (**a**) indicate the Pt/film/substrate interfaces. Pt layer was deposited on the top of the device to protect the film from ion beam damage during the sample preparation and to serve as an anode for IV measurements.

Figure 7 shows cross-section STEM-HAADF images of the device after performing the in-situ IV measurements. The Sr deficient MOVPE layer is visible by reduced Z-contrast compared to the stoichiometric substrate, the platinum contact is visible by its grainy structure.

Figure 7a–c show the device after cycling up to 5 V. No indications of filaments or foreign phases are visible in the SrTiO_3_ films. After applying higher voltages beyond 7 V (Fig. 7d) contrast fluctuations (bright and dark) are visible in the thin SrTiO_3_ film on the top of the cathode (substrate). Most pronounced is the feature indicted by an arrow, that appears at reduced HAADF intensity and resembles the appearance of conducting filaments as shown by Du et al.^47^ These in-situ STEM measurements corroborate our finding that the resistive switching in our Sr deficient SrTiO_3_ MOVPE thin film, which is observed at substantially lower voltages than those required to form filaments, does not involve filament formation. In contrast, our preliminary studies suggest an alternative scenario for resistive switching in Sr deficient films. For instance, Ti-antisite defects have been reported to be mid-gap defect states that induce ferroelectric polarization due to off-center position^5,48,49^. The resistive switching could then be based on trap assisted tunneling or ferroelectric switching. Focused investigations to identify the switching mechanism in our devices are ongoing and will be presented elsewhere.

## Summary

We have shown that phase-pure stoichiometric and off-stoichiometric SrTiO_3_ thin films can be grown on (100) SrTiO_3_:Nb substrates without extended defects by MOVPE. We took advantage of the independent control of the all components in the gas phase together with a high oxygen partial pressure in MOVPE and varied the composition of the gas phase by adjusting the (Sr/Ti)_liq_ precursor ratio. We have achieved Sr deficiency in the growing layer up to 20% without structural degeneration, as validated by TEM and HRXRD. In addition, to an increasing vertical lattice parameter *d*_*⊥*_, Sr deficiency increases the permittivity in SrTiO_3_, and triggers an intrinsic resistive switching effect with an on–off ratio of about 10^3^ – while no resistive switching was observed in stoichiometric thin films for the same measurement range. IV measurements at 10 K again showed resistive switching for an off-stoichiometric SrTiO_3_ MOVPE thin film, rendering ionic diffusion processes unlikely, and instead suggesting a defect-based mechanism. We show that the hysteresis evolves without forming pulse or any abrupt threshold. Moreover, the HRS after onset of switching shows a higher resistivity than the layer below the threshold. This suggests that mass transport by ion diffusion does not play an important role in the switching mechanism. Supportingly, in-situ TEM experiments clearly show the absence of filaments in the applied voltage regime.

## Methods

SrTiO_3_ thin films with thicknesses between 40 and 50 nm were epitaxially grown by means of liquid-delivery spin MOVPE^50^ on 0.5 wt% Nb-doped SrTiO_3_ (100) substrates (from CrysTec GmbH Berlin, Germany) and DyScO_3_ (110), both with 0.1° off-cut^51^. SrTiO_3_ substrates were pre-treated by etching with a buffered hydrofluoric acid solution and subsequent heating in pure oxygen atmosphere at 1100 °C for one hour. A surface with periodically arranged, atomically smooth ≈ 200 nm wide terraces with single unit cell high steps of ≈ 4 Å (TiO_2_ termination) is thereby formed^52,53^. Similarly terraced surfaces were achieved for DyScO_3_ substrates after cleaning the surface with acetone and deionized water and subsequent heating in pure oxygen for 10 h at 1050 °C^54^. Metal–organic (MO) precursors Ti(O^i^Pr)_2_(tmhd)_2_^55^ and Sr(tmhd)_2_-tetraglyme^56^ were dissolved in dry toluene and vaporized by two independent evaporation systems at T_v_ = 210 °C. The films were grown at varying temperatures ranging from 550 °C to 750 °C at 15 mbar chamber pressure with fluxes of 1500 sccm and 5000 sccm for argon and oxygen, respectively.

In the homoepitaxial films, the precursor concentration ratio in the source liquids (Sr/Ti)_liq_ was varied between 2.0 and 3.6 with an absolute Sr precursor concentration of 0.02 mol/l < c_Sr(tmhd)2_ < 0.03 mol/l by keeping the total precursor concentration constant. The carrier rotation was adjusted to be 600 rpm to ensure a homogeneous mixture of all gases, a laminar precursor-solvent-O_2_/Ar stream and a uniform supersaturation zone above the susceptor. Subsequently, the grown films were cooled down inside the reaction chamber with 10 K/min in 300 mbar O_2_ atmosphere by keeping up the carrier rotation constant. The SrTiO_3_ films were characterized by HRXRD performed in a Rigaku SmartLab diffractometer using a monochromatic (*λ* = 1.54056 Å) well collimated primary beam (asymmetric Ge(220) 2-bounce monochromator channel-cut crystal with incidence beam divergence of 12 arc. sec) and a HyPix-3000 horizontal 2D detector. In addition, STEM-HAADF and LAADF was performed in a FEI Titan 80–300 operating at 300 kV, which is equipped with a highly brilliant cathode (X-FEG). The semi-convergence angle was tuned to 9 mrad and the semi-acceptance angle of the detector was set to 35 mrad (camera length 196 mm) and 23 mrad (camera length 300 mm). STEM-EDX was carried out in a JEOL JEM2200FS operated at 200 keV equipped with an X-ray SD detector (Bruker). TEM samples were prepared by plan parallel polishing to a thickness of about 10 µm and final ion milling using a Gatan Pips with liquid nitrogen cooling.

In-situ STEM observations were performed in a same FEI Titan, as for ex-situ measurements above. Cross-sectional TEM specimens from as-grown device was prepared by focused ion beam (FIB) milling using the Thermo Fischer Scientific Nova 600 dual beam system. At around 200 nm thickness, thinning at 30 kV accelerating voltage was stopped to prevent the formation of an amorphous layer on both sidewalls of the specimen. Then, low kV cleaning is carried out at 5 kV and 2 kV to reach the thickness of 100 nm. The FIB lamella was mounted onto the Protochips Fusion 500 double-tilt holder for electrical measurements.

Electrical and dielectric properties of the homoepitaxial films were investigated at room temperature by preparing the following test structure. Circular platinum contacts of 20 nm thickness with diameters of 150 µm, 200 µm, and 300 µm were deposited through a shadow mask by electron beam evaporation on the as grown films. Thus a MOS test structure composed of a Pt/SrTiO_3_/SrTiO_3_:Nb stack was formed. For mechanical contact stability, the platinum was covered with additional 20 nm of nickel. Ohmic backside contacts were formed by liquid Ga/In eutectic. A Keithley 237 source measure unit was used for measuring IV characteristics and by a Boonton capacitance meter 7200 (test frequency 1 MHz, test level 100 mV) the zero-bias capacitance was determined. The measurements were performed in the dark to avoid effects caused by photocurrent or photocapacitance^57,58^.

## Supplementary Information

Supplementary Information.

## Data Availability

The data that support the findings of this study are available from the corresponding author upon reasonable request.
