# Supplementary material for: Influence of Sr deficiency on structural and electrical properties of SrTiO3 thin films grown by metal–organic vapor phase epitaxy
Source: Sci Rep. 2021 Apr 5;11:7497. doi: 10.1038/s41598-021-87007-2 (PMC8021553; doi:10.1038/s41598-021-87007-2)
Supplement: Supplementary file 1 — Supplementary Information. [file 41598_2021_87007_MOESM1_ESM.pdf]

# Influence of Sr deficiency on structural and electrical properties of $SrTiO_3$ thin films grown by metal-organic vapor phase epitaxy

Aykut Baki[a], Julian Stöver, Tobias Schulz, Toni Markurt, Houari Amari, Carsten Richter, Jens Martin, Klaus Irmscher, Martin Albrecht, and Jutta Schwarzkopf

*Leibniz-Institut für Kristallzüchtung, Max-Born-Straße 2, 12489 Berlin, Germany*

[a]aykut.baki@ikz-berlin.de

## Supplemental

*Simulation of HRXRD patterns*

The simulation of the HRXRD data presented in Figure 1b and 1c was performed by using open source X-ray utilities package.[S1] For the simulations, we used a lattice constant of $d_\perp$ = 3.905 Å for the $SrTiO_3$ substrate. To take into account the defective interface region between substrate and thin film, an offset of around 15 pm was assumed, following LeBeau.[S2]

Figure 6a shows an exemplary HRXRD pattern and the simulation of an off-stoichiometric film with $(Sr/Ti)_{liq}$ = 2.9. The resulting depth profile of the film is based on multiple sublayers revealing a highly inhomogeneous defect distribution. This is in-line with the observations from STEM-HAADF. For the simulation in Figure 6, we considered a stack of eight different sublayers each having a different vertical lattice parameter. The averaged value for the vertical lattice parameter $d_\perp$, which is indicated by the red dotted line in Figure 6b, perfectly fits to the HRXRD peak of the layer, as shown in Figure 1c and 1d (blue arrow). The mean lattice parameter was calculated as the arithmetic average of the eight vertical lattice parameters considering the individual layer thicknesses.

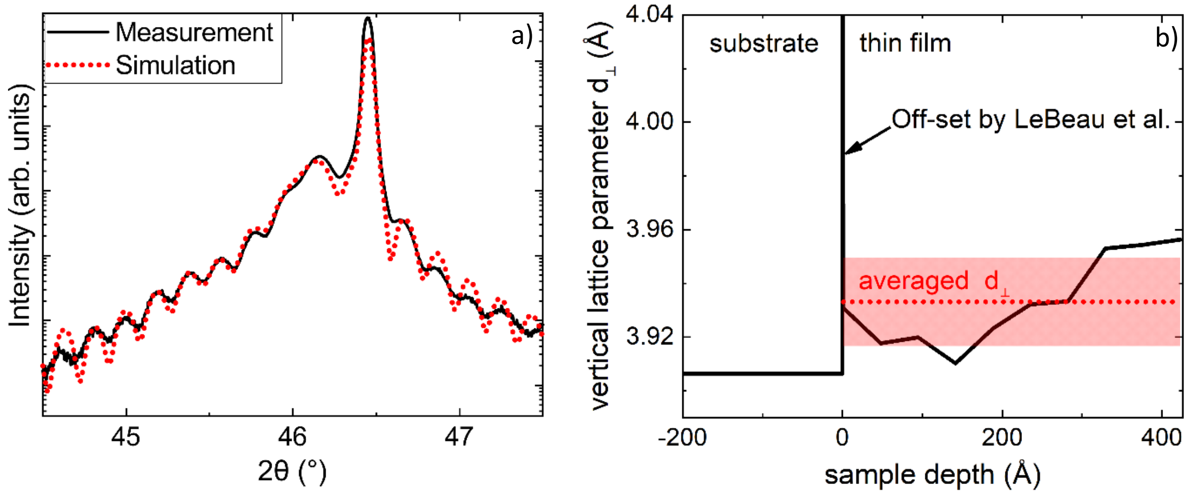


Figure S1: Simulation of HRXRD data of off-stoichiometric sample with $(Sr/Ti)_{liq}$ = 2.9. (a) HRXRD pattern

in the vicinity of the (200) $SrTiO_3$:Nb substrate peak – experimental (solid black) and simulated (doted red). (b) Applied depth profile including averaged vertical lattice parameter $d_{\perp}$ and error bar. The interface off-set analogues to LeBeau et al. is labeled by an arrow and basically illustrates a void of 15 pm between substrate and thin film.

For the stoichiometric film with $(Sr/Ti)_{liq}$ = 3.6, it suffices to consider only a single layer to reach an adequate fit of the film's HRXRD pattern. The HRXRD pattern in the vicinity of the (100) and (200) $SrTiO_3$:Nb substrate peak and their corresponding simulation are displayed in Figure 7a and 7b, respectively. For this layer the lattice parameter yields $d_{\perp}$ = 3.907 Å.

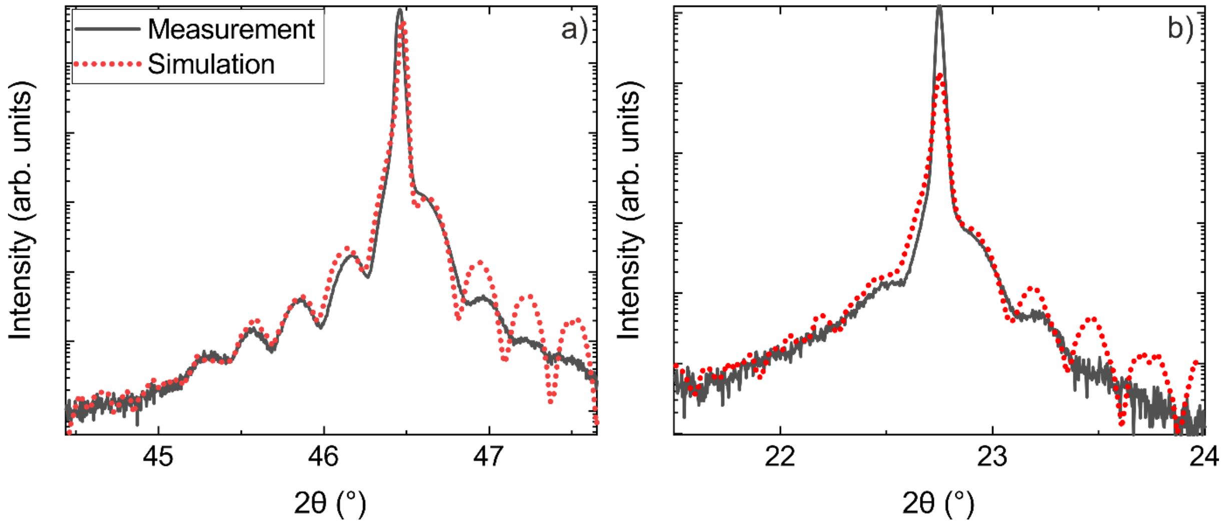


Figure S2: Simulation of HRXRD data of stoichiometric sample with $(Sr/Ti)_{liq}$ = 3.6. HRXRD pattern in the vicinity of the (a) (200) $SrTiO_3$:Nb substrate peak, and (b) (100) $SrTiO_3$:Nb substrate peak. Experimental and simulated data are presented by black and red, dotted lines, respectively.

*Sublayer structure*

Figures 8a and b shows two exemplary STEM ADF images of an off-stoichiometric sample with $(Sr/Ti)_{liq}$ = 3.2 acquired in the same region of the sample for two different camera lengths of 130 mm and 300 mm, respectively. The shorter camera length of 130 mm corresponds to HAADF conditions, which makes image intensity mainly reflect the atomic mass contrast (Z-contrast). On contrary, a higher camera length of 300 mm corresponds to low angle annular dark field (LAADF) conditions, diffusely scattered primary electrons are primarily contributing to the image intensity, and indicate local lattice distortions. For both imaging conditions, the MOVPE layer becomes distinguishable by means of a contrast change with respect to the $SrTiO_3$ substrate, confirming the presence of atomic defects within the layer. In detail, off-stoichiometric layers generally appear darker for HAADF conditions, implying a lower atomic mass density as compared to the stoichiometric substrate. Vice versa, the brighter intensity under LAADF conditions suggests that these underlying defects

additionally cause strong lattice distortions. Notably, the contrast in the MOVPE layer and thus the underlying defect distribution is inhomogeneous, particularly along the growth direction. In a rough estimation, this region may be divided into three sub-layers each having a different defect concentration. The reason for this inhomogeneous defect distribution within the layer is not clear at the moment, but explains the oscillations in the XRD pattern.

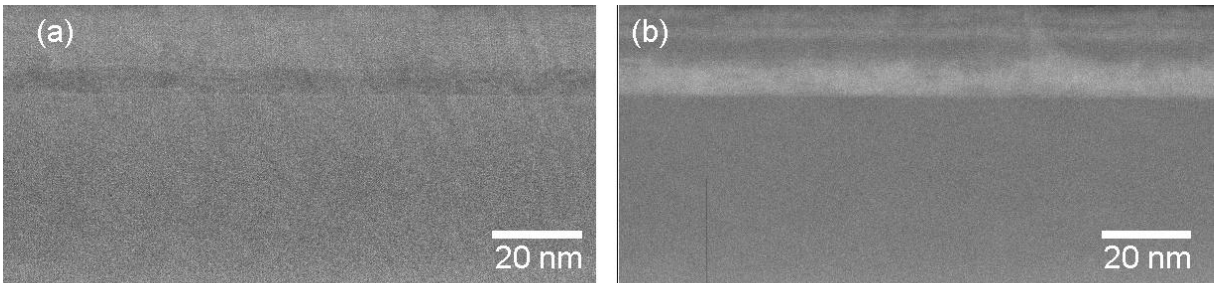


Figure S3: STEM LAADF and HAADF images of an off-stoichiometric $SrTiO_3$ layer with $(Sr/Ti)_{liq}$ = 3.2 for camera lengths of (a) 130 mm (HAADF) and (b) 300 mm (LAADF).

*Antisite domain boundary*

With increasing Sr deficiency, we observe a stacking defect in the MOVPE layer, as can be seen from the STEM-HAADF image in Figure 9. In the area marked as region A, the unperturbed $SrTiO_3$ stacking is observed, allowing the Ti and the Sr sublattices to be discriminated by the brighter appearance of the latter. In the defective region B, the STEM-HAADF pattern exhibits additional intensity spots in between the Sr positions of the unperturbed $SrTiO_3$ unit cell. Furthermore, the intensity spots related to the undisturbed Sr sublattice, as well as the additional intensities in between exhibit a comparable HAADF intensity, suggest that all columns exhibit similar mean Z number. We propose the following atomistic model to explain this effect as depicted in Figure 9b and c. Figure 9b shows the defective region B parallel to the e-beam direction. In the center of the structural model, the translation symmetry is broken by a shift of a half lattice constant perpendicular to the beam direction, resembling an antisite domain boundary. In the direction of the e-beam (Figure 9c), this results in an intermixing of the Sr and the Ti sublattices, causing additional spots in between the undisturbed Sr sites with all columns exhibiting an identical composition. Both observations again agree with our experimental observations.

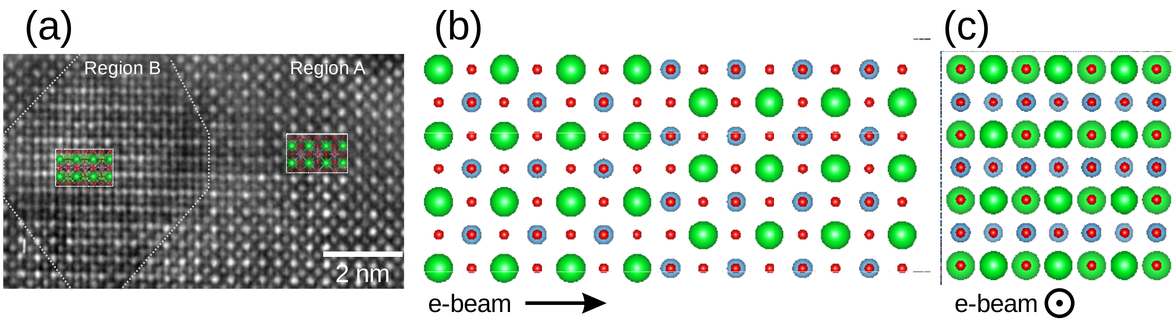


Figure S4: (a) STEM-HAADF image of $SrTiO_3$ thin film with $(Sr/Ti)_{liq}$ = 2.6, showing region A with regular $SrTiO_3$ STEM pattern and Region B where the translation symmetry. Sketch of the defective region B aligned (b) and (c) along the electron beam.

*Sr deficiency quantification*

To confirm a Sr deficiency up to 20 % as deduced from STEM EDX measurements, we performed STEM HAADF off-axis measurements as displayed in Figure 10a. Under these conditions, the HAADF intensity is less sensitive to local bending of the atomic columns. The HAADF intensity is plotted in Figure 10b along with a background fit. The decrease of the HAADF intensity in the region of the layer determines the loss of material up to 8% in lower part of the film. Using the molar masses of M(Sr) = 87.62 u, M(Ti) = 47.867 u, and M(O) = 15.999 u yields a molar mass for $SrTiO_3$ of 183.48 u. Therefore, a material loss of 8 % equals a molar mass of 165.14 u. The amount of Sr vacancies to reach this mass loss fraction is about 16 %. Considering the spatial inhomogeneities of the defect distribution within the MOVPE layer, such values thus confirm the results from STEM EDX.

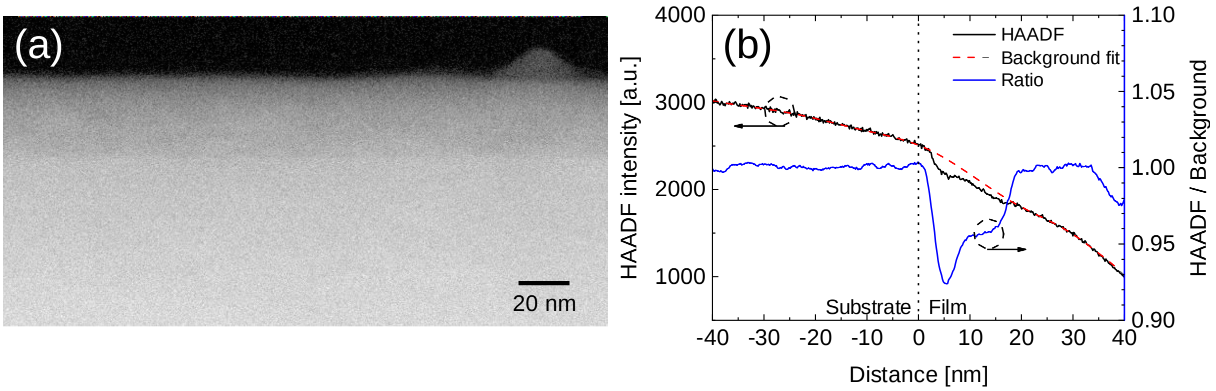


Figure S5: (a) STEM HAADF off-axis measurements for $SrTiO_3$ thin film with $(Sr/Ti)_{liq}$ = 3.2, (b) HAADF intensity in depth profile with a background fit.


S1. Kriegner, D., Wintersberger, E. & Stangl, J. Xrayutilities: A versatile tool for reciprocal space conversion of scattering data recorded with linear and area detectors. *J. Appl. Crystallogr.* **46**, 1162–1170 (2013).

S2. Lebeau, J. M. *et al.* Stoichiometry optimization of homoepitaxial oxide thin films using x-ray diffraction. *Appl. Phys. Lett.* **95**, 12–15 (2009).
